# Iba1 immunoreactivity is enhanced following an antigen retrieval treatment with EDTA, pH 6.0

**DOI:** 10.1016/j.mex.2014.10.007

**Published:** 2014-11-03

**Authors:** Atreyi Ghatak, Colin K. Combs

**Affiliations:** Department of Basic Science, University of North Dakota School of Medicine and Health Sciences, Grand Forks, ND 58203, United States

**Keywords:** Antigen retrieval, Iba1, Microgliosis, Immunostaining, Alzheimer's disease, Immunoreactivity

## Abstract

Antigen retrieval is a standard procedure to enhance immunohistochemical detection. However, among the many choices of techniques available for antigen retrieval, it is important to choose a method that works specifically for the antibody of interest. The small calcium binding protein, Iba1, has been well characterized as a microglia specific marker useful for identifying both resting and activated populations (Ito et al., 1998 [1]). In this study, we tested whether antigen retrieval methods would increase the sensitivity or improve the morphologic visualization of Iba1 immunoreactive microglia in the brains of wild type C57BL/6 mice and an APP/PS1 mouse model of Alzheimer's disease (AD). A more sensitive detection method might allow for better quantitation of microglial changes during disease. We modified a protocol which used three different methods and their combination for retrieving specifically anti-Aβ immunoreactivity in AD mouse brains to determine whether it improved Iba1 staining (Kai et al., 2012 [2]; Murayama et al., 1999 [3]). The following modifications were made to the original protocol:

1. We boiled the free floating brain sections or slide mounted brain sections in 10 mM EDTA solution (pH 6.0) in a secondary water bath instead of autoclaving for attempting Iba1 antigen retrieval.
2. We used a 15 min, 0.25% trypsin-EDTA treatment instead of protease K for attempting Iba1 antigen retrieval.
3. We immunostained with anti-Iba1 antibody as our primary interest, but also stained some sections in parallel with 4G8 antibody for anti-Aβ staining comparison.

Iba1 immunoreactivity was best enhanced by boiling in the low pH EDTA solution for both free floating and slide mounted tissues.

## Method details

As an approach to optimize immunostaining of Iba 1, a resting and activated microglial marker [1], we compared three different individual methods and a combination that used all three, for antigen retrieval on mouse brain tissue based on a protocol previously optimized for Aβ immunostaining [2], [3]. Although our intent was to develop a protocol for enhancing Iba1 immunoreactivity, we compared Aβ immunoreactivity in parallel.

### Tissue collection

1. *Animals*: Male wild type littermate control (C57BL/6) and male APP/PS1 [strain 005864 B6.Cg-Tg (APPswe, PSEN1dE9) 85Dbo/Mmjax, Jackson Laboratory] mice aged 9–10 months and 13 months were used for this study.
2. *Preparation of tissue*: Brains of wild type (WT) and APP/PS1 mice aged 9–10 months were collected and post-fixed in 4% paraformaldehyde, pH 7.4 for 7 days and then changed to 30% sucrose in PBS for 14 days changing the sucrose every 4 days for cryoprotection. The cryoprotected brains were frozen onto a Physitemp BFS-MP freezing stage set at −30 °C (Clifton, NJ) using Tissue Freezing Media (TFM-5, Triangle Biomedical Science, Inc., Durham, NC) that was mounted onto a Reichart OME sliding microtome. The frozen brains were serially sectioned, 40 μm, using the sliding microtome into six well tissue culture plates containing PBS with 0.02% sodium azide for later immunostaining.

Brains from 13 month old WT and APP/PS1 mice were also post-fixed in 4% paraformaldehyde, pH 7.4 for 7 days then changed to 30% sucrose in PBS for 14 days. The sucrose solution was replaced every 4 days. After this step, the sucrose saturated, cryoprotected brains were placed into a small hand-made mold made from aluminum foil that was approximately the size of each brain. The brains in the molds were then covered with Tissue Freezing Media (TFM-5) and flash frozen in liquid nitrogen by immersion of the foil mold. The frozen, embedded brains were then quickly frozen onto the cold stage (chuck) of a Leica CM1850 cryostat (Buffalo Grove, IL) set at −20 °C using additional Tissue Freezing Media. Serial, 10 μm, sections were cut and directly mounted onto gelatin coated subbed glass slides for later immunostaining.

### Antigen retrieval (AR) methods

#### Proteolytic digestion with trypsin

Free floating tissue sections were incubated in trypsin-EDTA (0.25% Trypsin, 2.21 mM EDTA, Corning Cellgro) in closed 5 mL Eppendorf plastic tubes for 15 min in a 37 °C incubator immediately followed by rinsing in room temperature ultrapure Type I water for 5 min and another incubation in ultrapure Type I water for 5 min at room temperature. Utmost care was taken during the wash steps to ensure that the tissue sections were not disturbed. The tubes were not rocked during rinses and were simply left on a laboratory bench. Only the respective solutions were carefully discarded via pipette removal at each wash step leaving the tissue sections at the bottom of the tubes.

#### EDTA (pH 6.0) boiling

Free floating tissue sections were submerged in a 10 mM EDTA, pH 6.0 solution in closed 5 mL Eppendorf plastic tubes. These were placed into a floating foam tube rack and transferred to a 500 mL glass beaker with boiling water on a hot plate for 10 min. After boiling, the tissue sections were immediately rinsed with room temperature ultrapure Type I water for 5 min followed by two rinses in ultrapure Type I water for an additional 5 min. The tubes were not rocked during rinses and were simply left on a laboratory bench. Again, care was taken to remove the solution during rinses without disturbing the sections resting on the bottom of the tubes.

For slide mounted tissue sections, the slides were placed inside a closed glass Coplin jar filled with the EDTA solution (pH 6.0). The sealed Coplin jar was placed inside a 500 mL glass beaker filled with boiling water on a hot plate for 10 min. Similar to the procedure for free floating sections, after boiling the slides were immediately rinsed with room temperature ultrapure Type I water for 5 min followed by two successive rinses in ultrapure Type I water for an additional 5 min.

#### Formic acid treatment

In a chemical fume hood, free floating tissue sections were incubated in 98% formic acid (EMD) inside closed 5 mL Eppendorf plastic tubes for 5 min at room temperature followed by removal for chemical waste disposal. The sections were immediately rinsed with room temperature ultrapure Type I water for 5 min followed by two additional 5 min incubations in ultrapure Type I water. The tubes were not rocked during rinses and were simply left on a laboratory bench. Care was taken again to remove solutions from the tubes during rinsing without disturbing tissue at the bottom of the tubes.

#### Combination treatment method

This was referred to as the PFA combination method in the original protocol that we modified to substitute a trypsin-EDTA treatment for protease K. The combination method employed all three methods of AR described above in the order listed. Free floating tissue sections first went through trypsin-EDTA digestion followed by EDTA boiling, and then formic acid treatment. Each of the treatments was followed by two, 5 min rinses in ultrapure Type I water at room temperature, identical to the described procedure in the antigen retrieval steps described above.

### Immunostaining

After antigen retrieval methods, free floating tissue sections were transferred to plastic mesh 70 μm cell strainers inserts (352350, BD Falcon, Franklin Lakes, NJ) and placed into six well Cellstar plastic cell culture dishes (657-160, Greiner Bio-one, Monroe, NC) without lids for immunostaining. Use of the mesh bottom cell strainers allowed these to be picked up with metal forceps to easily pipette out underlying solutions in the wells during the various rinses and incubations described below. Slide mounted sections were laid flat into a Prohisto slide staining container (Columbia, SC) for immunostaining. Use of the Prohisto container allowed each slide to be in an individual well for simple pipette addition or removal of solutions for the various rinses and incubations described below. All rinses and incubations were performed by placing the six well plates of free floating tissue onto a 55S Single Platform Shaker set at 10 rocking motions per minute (Reliable Scientific, Inc., Nesbit, MS) for the times indicated below. The slide mounted tissue inside the Prohisto Slide container was left on the benchtop for all rinsing or incubating steps without any rocking.

All free floating tissue sections or slide mounted tissue sections were briefly washed in PBS for 5 min and then incubated with 0.3% H_2_O_2_ in PBS for 5 min at room temperature to inhibit endogenous peroxidase activity. This was followed by an immediate wash with PBS for 5 min.

For anti-Iba1 staining, free floating tissue sections or slide mounted tissue sections were blocked in PBS solution (PBS with 0.5% bovine serum albumin, BSA, 5% goat serum, and 0.1% Triton X-100) for 1 h, and then incubated in primary antibody in PBS solution (1:1000 anti-Iba1, rabbit polyclonal, 019-19741, Wako Pure Chemicals) overnight at 4 °C. After primary antibody incubation, the free floating tissue or slide mounted tissue sections were rinsed four times, 10 min each (with the PBS solution), and then incubated with biotinylated anti-rabbit secondary antibody (1:2000, Vector Laboratories) for 2 h at room temperature. The secondary antibody was removed and the free floating tissue or slide mounted tissue sections were rinsed four times with PBS solution, and then incubated with PBS-Avidin-Biotin solution (2 μL each of solutions A and B per mL of PBS, Vectastain ABC kit, Vector Laboratories) for 2 h at room temperature. The free floating tissue or slide mounted tissue sections were rinsed four times with PBS only, then incubated for 5 min with a Vector VIP chromogen solution according to manufacturer's instructions (Vector VIP peroxidase substrate kit, Violet SK-4600, Vector Laboratories) to develop the immunostains.

For anti-Aβ staining, free floating tissue sections were blocked for an hour with M.O.M.™ mouse Ig blocking reagent (M.O.M. kit, Vector Laboratories) prepared in regular PBS according to the manufacturer's protocol. The sections were then incubated in primary antibody (1:1000 anti-β amyloid, 4G8, Sig 39220, Covance) made up in PBS solution overnight at 4 °C. All the steps thereafter were identical for anti-Iba1 staining described above except an anti-mouse biotinylated secondary antibody (1:9000, Vector Laboratories) was substituted for the secondary antibody incubation step. Free floating sections were mounted onto gelatin subbed glass slides and allowed to dry overnight. Slide mounted sections were also allowed to dry overnight.

Since all stained sections (both free floating and slide mounted) were now on gelatin subbed slides, they were treated the same for dehydration, clearing, and coverslipping. Slides were first serially dehydrated through Coplin jar rinses in distilled water, 50% ethanol, 70% ethanol, 95% ethanol, and 100% ethanol for 5 min each. The sections were then defatted (cleared) through two rinses in Coplin jars, 5 min each, using Histoclear (National Diagnostics, Atlanta, GA). The slides were immediately coverslipped using glass coverslips and Permount Mounting Media (Fisher Scientific, Fairlawn, NJ) and allowed to dry overnight.

All slides were carefully examined using an upright Leica DM1000 microscope. Images were captured using a DFC 320 digital camera from the temporal cortex region of the brains using LAS V4.2 software (Leica microsystems). Figures were made using Adobe Photoshop CS5 (Adobe systems).

Out of the methods used, boiling the tissue in EDTA, pH 6.0 (Fig. 1e and f) resulted in improved immunodetection of microglia (anti-Iba1) in both WT and APP/PS1 brains in free floating sections compared to corresponding no retrieval method controls (Fig. 1a and b). Trypsin digestion, formic acid treatment, and the combined treatments all decreased Iba1 immunoreactivity compared to no treatment controls (Fig. 1c, d, g, h, i, j). Based upon this observation, only the 10 mM EDTA, pH 6.0 antigen retrieval was attempted on slide mounted sections. As in free floating sections, there was a clear increase in not only Iba1 immunostaining intensity but also visualization of microglial morphology in the antigen retrieved tissue (Fig. 2c and d) compared to no treatment controls (Fig. 2a and b).

**Fig. 1:**
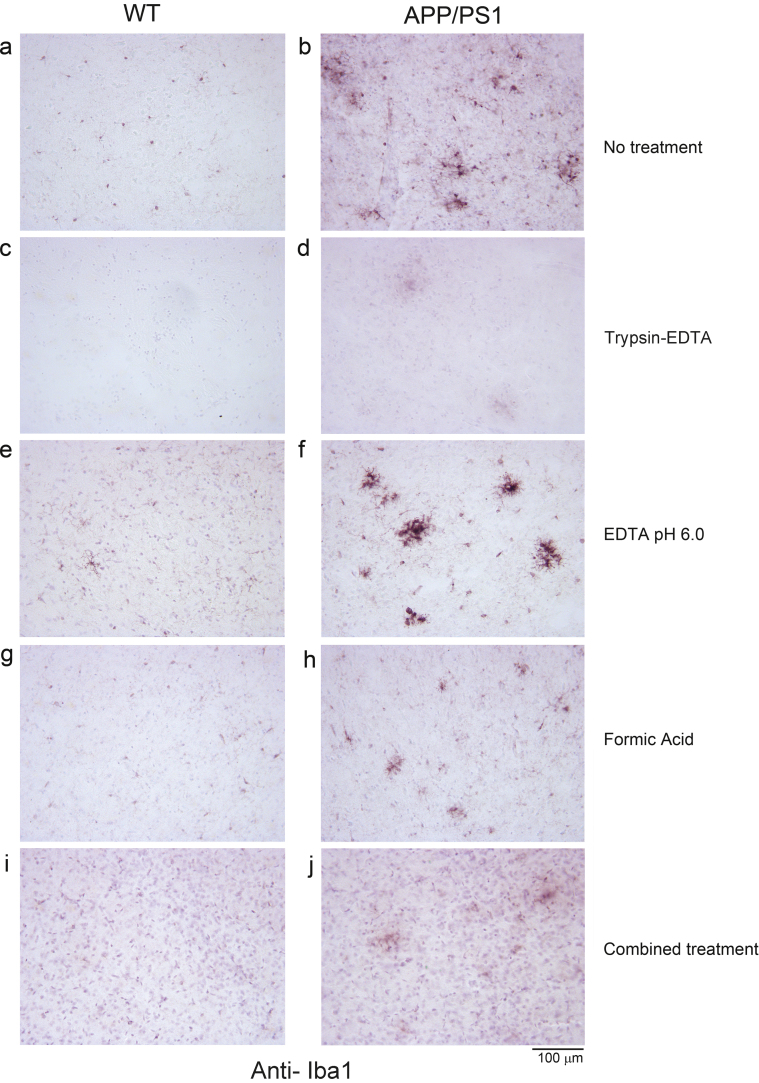
Iba1 immunostaining of free floating sections following different antigen methods. Wild type (WT) and APP/PS1 free floating 40 μm tissue sections were untreated (no treatment) or processed through single or combined antigen retrieval methods (trypsin-EDTA, 10 mM EDTA pH 6.0, formic acid, combined treatment) before immunostaining using anti-Iba1 antibody. Representative Iba1 immunostained images visualized using vector VIP as the chromogen taken from the temporal cortex are shown from (a) wild type (WT) no treatment control, (b) APP/PS1 no treatment control, (c) WT trypsin-EDTA antigen retrieval, (d) APP/PS1 trypsin-EDTA antigen retrieval, (e) WT 10 mM EDTA, pH 6.0 antigen retrieval, (f) APP/PS1 10 mM EDTA, pH 6.0 antigen retrieval, (g) WT formic acid antigen retrieval, (h) APP/PS1 formic acid antigen retrieval, (i) WT combined treatment antigen retrieval, and (j) APP/PS1 combined treatment antigen retrieval.

**Fig. 2:**
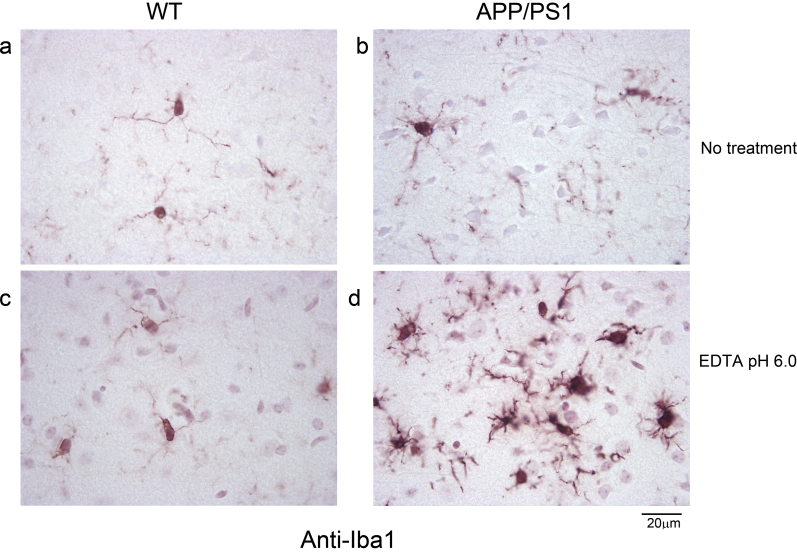
Morphology of Iba1 immunoreactive microglia on slide mounted sections visualized after 10 mM EDTA, pH 6.0 antigen retrieval. Wild type (WT) and APP/PS1slide mounted brain sections, 10 μm, were untreated (no treatment) or processed for antigen retrieval using 10 mM EDTA, pH 6.0 before immunostaining using anti-Iba-1 antibody and visualized using vector VIP as the chromogen. Representative temporal cortex images are shown from (a) Wild type (WT) no treatment control, (b) APP/PS1 no treatment control, (c) WT 10 mM EDTA, pH 6.0 antigen retrieval, and (d) APP/PS1 10 mM EDTA, pH 6.0 antigen retrieval conditions.

On the other hand, only formic acid (Fig. 3h) and the combined treatment (Fig. 3j) of the antigen retrieval protocols improved Aβ immunoreactivity, demonstrating that antigen selective retrieval methods are necessary for antibody-antigen combinations of interest.

**Fig. 3:**
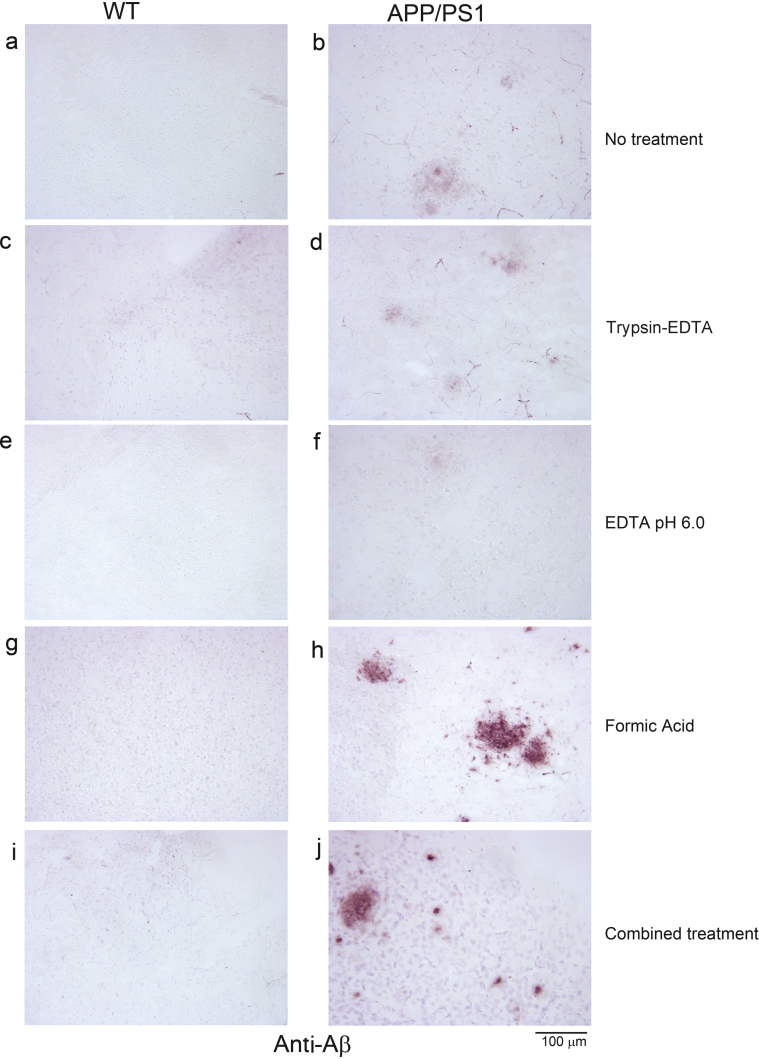
Aβ immunostaining of free floating sections following different antigen retrieval methods. Wild type (WT) and APP/PS1 free floating 40 μm tissue sections were untreated (no treatment) or processed through single or combined antigen retrieval methods (trypsin-EDTA, 10 mM EDTA pH 6.0, formic acid, combined treatment) before immunostaining using anti-Aβ antibody. Representative Aβ immunostained images visualized using vector VIP as the chromogen taken from the temporal cortex are shown from (a) wild type (WT) no treatment control, (b) APP/PS1 no treatment control, (c) WT trypsin-EDTA antigen retrieval, (d) APP/PS1 trypsin-EDTA antigen retrieval, (e) WT 10 mM EDTA, pH 6.0 antigen retrieval, (f) APP/PS1 10 mM EDTA, pH 6.0 antigen retrieval, (g) WT formic acid antigen retrieval, (h) APP/PS1 formic acid antigen retrieval, (i) WT combined treatment antigen retrieval, and (j) APP/PS1 combined treatment antigen retrieval.
